# Congenital neutropenia: disease models guiding new treatment strategies

**DOI:** 10.1097/MOH.0000000000000696

**Published:** 2021-11-24

**Authors:** Ivo P. Touw

**Affiliations:** Department of Hematology, Erasmus University Medical Center, Rotterdam, The Netherlands

**Keywords:** acute myeloid leukemia, congenital neutropenia, disease models, genome editing, growth factor therapy, leukemia predisposition, myelodysplasia

## Abstract

Myeloid diseases are often characterized by a disturbed regulation of myeloid cell proliferation, survival, and maturation. This may either result in a severe paucity of functional neutrophils (neutropenia), an excess production of mature cells (myeloproliferative disorders) or in clonal expansions of dysplastic or immature myeloid cells (myelodysplasia and acute myeloid leukemia). Although these conditions can be regarded as separate entities, caused by the accumulation of distinct sets of somatic gene mutations, it becomes increasingly clear that they may also evolve as the prime consequence of a congenital defect resulting in severe neutropenia. Prominent examples of such conditions include the genetically heterogeneous forms of severe congenital neutropenia (SCN) and Shwachman-Diamond Syndrome. CSF3 treatment is a successful therapy to alleviate neutropenia in the majority of these patients but does not cure the disease nor does it prevent malignant transformation. Allogeneic stem cell transplantation is currently the only therapeutic option to cure SCN, but is relatively cumbersome, e.g., hampered by treatment-related mortality and donor availability. Hence, there is a need for new therapeutic approaches.

**Recent findings:**

Developments in disease modeling, amongst others based on induced pluripotent stem cell and CRISPR/Cas9 based gene-editing technologies, have created new insights in disease biology and possibilities for treatment. In addition, they are fueling expectations for advanced disease monitoring to prevent malignant transformation.

**Summary:**

This review highlights the recent progress made in SCN disease modeling and discusses the challenges that are still ahead of us to gain a better understanding of the biological heterogeneity of the disease and its consequences for patient care.

## INTRODUCTION

Hereditary bone marrow (BM) failure syndromes prone to the development of myeloid malignancies such as myelodysplastic syndrome (MDS) and acute myeloid leukemia (AML) have been the subject of study for many decades. The introduction of advanced genetics based on next generation sequencing technologies has advanced our understanding of the biology of these conditions and has improved diagnosis, prognostication and disease monitoring [1,2]. Likewise, genomic studies in large adult and pediatric populations, which identified germ-line vulnerabilities for leukemia predisposition in hematologically healthy subjects, have resulted in a better understanding of how malignancies arising in these cases should be prognostically classified and treated [3^▪^].

Severe congenital neutropenia (SCN) comprises a rare group of genetically heterogeneous congenital disorders characterized by a block in neutrophil differentiation in the BM, resulting in absolute neutrophil counts below 0.5 × 10^5^/L [4]. The most frequent genetic subtype of SCN, comprising up to 45% of newly diagnosed cases of SCN with a known mutation, is the autosomal dominant form characterized by mutations in *ELANE*, which encodes the azurophilic granule serine protease neutrophil elastase (NE). Mutations in *HAX1*, encoding a multifunctional protein amongst others involved in mitochondrial stability are the cause of the autosomal recessive condition originally known as Kostmann's disease. Other rare genetic subtypes include X-linked neutropenia with mutations in *WAS*, and cases with mutations in *GFI1*, *JAGN1*, *SLC37A4* and *VPS13B*[4]. Although Shwachman-Diamond Syndrome (SDS) is clinically distinguishable from other SCN forms, it is often classified as a form of SCN based on its severe shortage of neutrophils [4].

There is still a significant portion of SCN patients in which a genetic cause has not been identified [5]. Given the wide-variety of cellular defects incited by the mutations and the fact that they affect both cell intrinsic as well as nonintrinsic (‘niche’) components of the hematopoietic system raises fundamental questions of how these defects cause neutropenia, and if so, to what extent they affect overlapping or distinct molecular mechanism driving neutrophil development. This obviously has ramifications for finding unifying approaches aimed at curing these conditions. Although CSF3 therapy is the mainstay in the treatment of SCN patients to alleviate severe neutropenia and to prevent infection-related mortality and morbidity, contra-indications include poor neutrophil recovery, even when high dosages are given on a daily basis, and the risk of malignant transformation in some genetic subtypes of SCN. The clinical management of chronic neutropenia requires a careful evaluation and sustained monitoring of the severity of symptoms including bacterial and viral infections, fever, changes in CSF3 dose requirements, signs of progression to malignancy, etc., for which updated decision algorithms based on expert's opinions have recently become available [4,6]

Currently, more than 25 genes associated with congenital neutropenia have been identified, not necessarily leading to its most severe forms [4]. Still, new genetic subtypes of SCN/SDS are being identified, most recently pedigrees with mutations in *CLPB*, a gene encoding a protein that localizes to mitochondria and regulates mitochondrial function [7,8], *SEC61A1* involved in endoplasmic reticulum (ER) protein transport and calcium homeostasis and *SRP54*, encoding a protein constituent of a ribonucleoprotein complex involved in translocation of nascent polypeptides to the ER surface [9,10]. With the list of genes causing SCN still growing, it becomes increasingly possible to classify SCN not only based on the type of inheritance and disease features, but also on the type of cellular defects caused by the mutations [4,6]. 

**Box 1:**
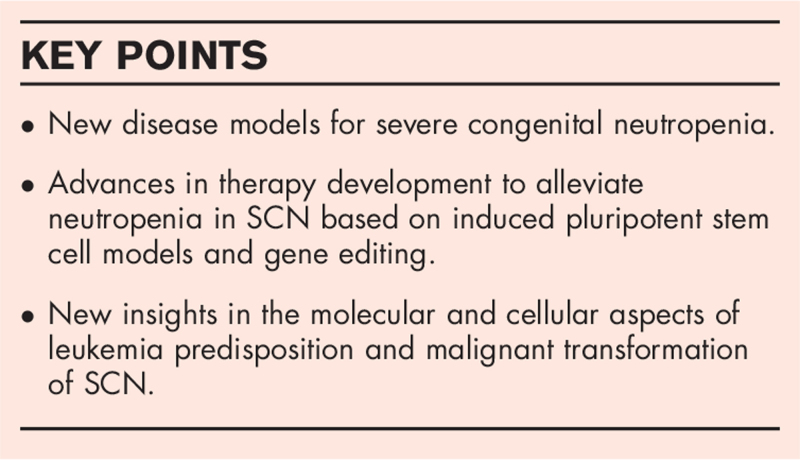
no caption available

## ADVANCES IN DISEASE MODELING

It continues to be a challenge to pinpoint how the wide spectrum of mutations in SCN cause defective neutrophil production. This particularly applies to ELANE-SCN, in which the *ELANE* mutations are spread out over the entire exome and sometimes even occur in noncoding regions [11–13]. Although multiple mechanisms causing neutropenia have been postulated, none of these have so far been firmly linked to specific *ELANE* mutations [14^▪^]. This is mainly due to the fact that mouse models with pathognomonic *ELANE* mutations are not neutropenic, suggestive of crucial species differences between mice and humans [4,14^▪^]. A similar discrepancy exists for mouse models of HAX1-SCN and other genetic subtypes [4]. In contrast, mice with patient-derived *GFI1* mutations are neutropenic and provide an attractive model for studying the impact of these mutations on neutrophil development in depth. Intriguingly, by using single cell ATAC-seq, Muench and colleagues showed that the consequences of *GFI1* mutations on transcription of target genes are not static, but dynamic, i.e., sequentially altering as the myeloid progenitors go through successive stages of differentiation [15]. Although still mostly hypothesis-generating and restricted to a single genetic subtype of SCN, these data fuel the concept that dynamic, cell-state-dependent effects of mutations may affect the outcome of treatments and may vary between patients [15]. Conceivably, this could explain why patients with identical or similar mutations in *ELANE* may show differences in phenotype, most notably severe chronic versus cyclic neutropenia, and variations in responses to CSF3 therapy [14^▪^].

Recently, zebrafish models of HAX1-SCN, SDS and *SRP54* mutant, CSF3R and AK2-deficient disease have become available, which recapitulate the major disease features of patients and thus provide convenient settings for in vivo drug testing [10,16–21]. For instance, based on the findings in the ak2-deficient zebrafish model, it was suggested that antioxidants may have a therapeutic effect in AK2-deficient neutropenia patients [20]. On the other hand, zebrafish and mouse models for GATA2 deficient neutropenia show more subtle and complex defects suggestive of a chronic form of stress gradually resulting in myeloid phenotypes with advanced ageing [22–24].

## INDUCED PLURIPOTENT STEM CELL MODELS

The discovery of human induced pluripotent stem cells (iPSCs) revolutionized the field of developmental biology and opened unprecedented possibilities for disease modeling [25]. Patient-derived iPSCs have been generated for different genetic subtypes of SCN and SDS, which serve as attractive tools for dissecting the biological consequences of the disease causative mutations on neutrophil development from the committed myeloid progenitors onward. Although the earliest investigations mainly focused on model generation and validation [26,27], ongoing studies deal with elucidating the mechanistic aspects of severe neutropenia and to find new avenues for treatment. In addition, the patient-derived iPSC models have been employed to model the sequential steps of malignant transformation.

HAX1-SCN iPSC recapitulate the major features described in patients including disruption of the mitochondrial membrane potential leading to increased apoptosis [26,28,29^▪▪^,^30^]. SCN-iPSC lines with distinct *ELANE* mutations show a reduced but variable neutrophilic differentiation potential upon exposure to CSF3, likely reflecting the heterogeneous clinical responses to CSF3 treatment. [29^▪▪^,^31^–^35^]. These lines provide unique opportunities to unravel the biological consequences of different *ELANE* mutations in myeloid progenitors and their progeny, which has thus far been largely elusive [11]. Based on observations in primary patient samples, studies mainly aimed at the role of the unfolded protein response (UPR) and increased apoptosis in *ELANE*-mutant myeloid progenitor cells as the cause of neutropenia [36–38]. Because evidence for an elevated UPR was also obtained in other genetic subtypes of congenital neutropenia, this is considered a common albeit not a universal patho-mechanism of neutropenia [7,9,39,40]. However, not all *ELANE* mutations result in protein misfolding, leaving room for alternative explanations as to how *ELANE* mutations affect neutrophil development [41]. Studies in the iPSC models may shed further light into the heterogeneity of cellular responses of hematopoietic progenitor cells (HPCs) in ELANE-SCN. Using a protein structure prediction algorithm [42], it was recently shown that the majority of *ELANE* mutations in the currently generated SCN-iPSCs are predicted to result in protein misfolding [43]. Nonetheless, these lines did not uniformly show induction of the classical UPR, because often only a few UPR related transcripts were found to be upregulated, whereas others were unchanged or even downregulated [31,34,44].

As an alternative pathway for the classical UPR, the promyelocytic leukemia protein (PML) has been reported to interact with and degrade misfolded proteins. This particularly occurs under conditions of excessive oxidative stress and elevated production of reactive oxygen species, leading to the generation of PML nuclear bodies (PML-NBs) [45]. A recent study revealed that ELANE-misfolding mutations induce the formation of PML-NBs in the CD34^+^CD45^+^ HPCs derived from the iPSCs as well as in primary BM derived CD34^+^ Hematopoietic stem progenitor cells (HSPCs) from ELANE-SCN patients [29^▪▪^]. Importantly, mutant ELANE was highly expressed in these cells, which was not seen in HAX1-SCN or in ELANE-SCN without predicted misfolding mutations. Intriguingly, deletion of PML by CRISPR/Cas9 resulted in the loss of ELANE expression and restored the response of the HPCs to CSF3 in colony forming assays. Taken together, these results suggest that the lack of neutrophil recovery in response to CSF3 therapy in certain ELANE-SCN cases, particularly those with misfolding mutations, may be attributable to elevated PML expression as a result of excessive oxidative stress in the HSPC compartment [29^▪▪^].

SDS-iPSCs recapitulate the essential features of the disease such as defects in exocrine pancreatic and hematopoietic differentiation, enhanced apoptosis and elevated release of proteases [27,46]. Recently, it was shown that predisposition to apoptosis in the hematopoietic system extends to hemoangiogenic progenitors, hence not only resulting in neutropenia but also affecting the development of endothelial cells (EC) [47]. Finally, an iPSC model for reticular dysgenesis, like ELANE-SCN and HAX1-SCN characterized by a myeloid maturation arrest at the promyelocyte stage caused by mutations in *AK2*, showed that the mutation caused excessive oxidative stress in HSPCs. This could be reversed by antioxidant treatment, which restored differentiation of the AK2-deficient iPSCs into mature granulocytes [20].

## DEVELOPMENT OF NEW TREATMENT STRATEGIES

Although SCN patients are effectively treated by administration of recombinant CSF3, better known as granulocyte colony stimulating factor (G-CSF), neupogen or filgrastim, SCN patients face an increased risk of progression toward MDS or AML, especially when high dosages of CSF3 are needed to alleviate neutropenia [48,49]. The concern that CSF3 therapy might contribute to the leukemic progression is further supported by the finding that SCN patients often acquire truncating mutations in the G-CSF receptor (CSF3R) early during disease progression, eventually leading to the clonal outgrowth of malignant cells [4,50,51].

iPSC models hold potential for the identification of treatment strategies as alternatives for CSF3 therapy, i.e., to avoid development of malignancies and as options for SCN patients who fail to respond to CSF3. For example, one study showed that Wnt3a combined with CSF3, but not alone, stimulated the maturation of neutrophils in ELANE-C223X iPSC-derived hematopoietic cells. This suggested that, by using the combination therapy including compounds activating the Wnt3a/ß-catenin axis, CSF3 doses can be lowered in patients, thus potentially reducing the risk of leukemic transformation [44]. However, given the complexity of modulating Wnt signaling in vivo, it is not evident how this observation can conveniently be translated into a clinical protocol.

Another new treatment option that has been proposed is the use of the NE-specific cell-permeant serine protease inhibitor Sivelestat. Sivelestat, in combination with 50 ng/ml CSF3, mimicked high dose CSF3 treatment and ameliorated endoplasmic reticulum (ER) stress and enhanced cellular survival in ELANE-Q97P and ELANE-I118N mutant cells [34]. However, no such effects of Sivelestat were seen in a second study, which on the other hand showed that an alternative serine protease inhibitor, the β-lactam-based inhibitor MK0339, significantly increased the proportion of mature neutrophils in both ELANE-P139L, ELANE-G214R and control iPSC [32]. It remains unclear how these inhibitors exert their activities on the wide spectrum of mutant ELANE proteins and whether they can be clinically applied to alleviate neutropenia in SCN patients.

In iPSC models of SDS, hyperactive TGFβ signaling contributes to defective myeloid development, which can partly be restored by adding a TGFβ receptor small molecule inhibitor [52]. Another finding that holds therapeutic potential relates to the discovery of indirect somatic genetic rescue (SGR) involving acquired mutations in the *EIF6* gene, causing the loss of eIF6 protein in a subset of SDS patients [53^▪▪^,^54^^▪▪^]. It was elegantly shown in these papers that the loss of EIF6 protein compensates the ribosome assembly defect caused by the loss of the Shwachmann-Bodian-Diamond Syndrome protein and restores ineffective hematopoiesis by increasing fitness of the SDS deficient HSPCs. In contrast to mutations in p53, which arise in SDS HPSC and contribute to leukemic progression [50], SGR by *EIF6* mutations seems to have limited leukemic potential, thus opening a possible role for eIF6 suppressor mimics as a therapeutic strategy in SDS [54^▪▪^].

Conceivably the most promising therapeutic options to restore normal hematopoiesis in congenital neutropenia involve CRISPR-Cas9 based gene correction of HSPCs, combined with autologous stem cell reinfusion. This is particularly challenging in ELANE-SCN, given the wide variety of mutations. To cope with this, one strategy aims at deleting the mutant *ELANE* allele accepting that the wild-type allele will also be deleted, resulting in complete loss of NE expression [55,56^▪▪^]. This is considered acceptable given that patients suffering from Papillon-Lefevre syndrome, who lack functional NE and other neutrophil serine proteases, present with relatively mild symptoms [57]. A promising alternative approach aimed at mutant *ELANE* gene correction without losing NE expression has recently been developed using a repair template spanning the entire exon 4, which covers approximately 34% of the currently known *ELANE* mutations [58^▪▪^]. Notably, off-target mutations were undetectable, indicating that the procedure may eventually qualify for safe application in patients [58^▪▪^].

Although altogether fueling expectations, it is reasonable to state that the advances in therapeutic options to alleviate neutropenia based on disease modeling and genome editing are still relatively modest and await systematic and collaborative efforts from clinicians/pediatricians and clinical-translationally oriented researchers to further develop and implement these approaches into clinical protocols. Until this has been achieved, CSF3 therapy (or allogeneic stem cell transplantation in rare CSF3 nonresponsive cases) remains the prime treatment to avoid life-threatening infections in SCN.

## LEUKEMIC PROGRESSION MODELS OF SEVERE CONGENITAL NEUTROPENIA

Mutations in *CSF3R* are frequently observed in ELANE-SCN and HAX1-SCN patients who progress to MDS or AML. These mutations most often cause an intracellular truncation of the CSF3R protein leading to significant alterations in signaling behavior and can arise in minority clones years before leukemic progression occurs [4,51]. Once MDS or AML becomes overt, the malignant clones most frequently acquired mutations in *RUNX1* in clones already harboring *CSF3R* mutations [59].

**FIGURE 1:**
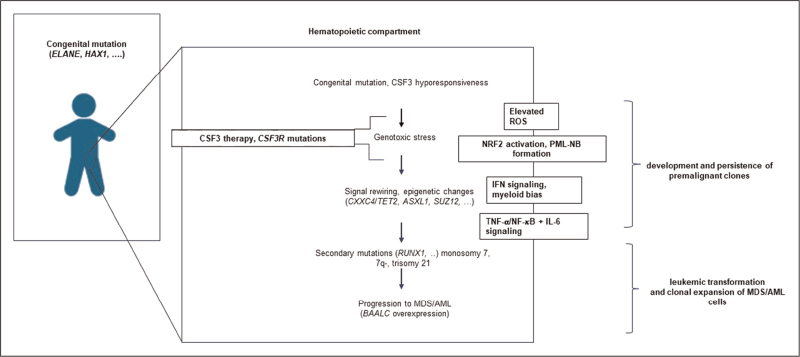
Model of leukemic progression of SCN. SCN, severe congenital neutropenia.

Because mouse models do not recapitulate the neutropenic phenotype seen in ELANE-SCN and ELANE-SCN patients, their value as leukemia predisposition models is restricted. Nonetheless, mice can be of value used to study the in vivo consequences of the acquired mutations involved in the leukemic transformation of SCN. For instance, such a mouse model was used to study the effects of *CSF3R* and *RUNX1* mutations and sustained CSF3 treatment on leukemic progression [60^▪^]. Mice with the combination of *CSF3R* and *RUNX1* mutations in their HSPCs exactly as those identified in a case of SCN/AML displayed an excess of immature myeloblasts (>10%) in the peripheral blood after 3 weeks of CSF3 treatment, which was not seen in the absence of either of the mutations or without CSF3 treatment [60^▪^,^61^]. Whereas excessive peripheral myeloblasts are generally considered a defining criterion of leukemia, these mice did not succumb to, or showed the moribund features associated with, AML. However, a re-transplantable AML occurred upon transplantation of the HSPCs from these mice in wild-type recipients, which was linked to the acquisition of a mutation in *CXXC4* and reduced expression of TET2 protein. Comparative RNAseq analysis of the mouse AML and the patient SCN/AML cells revealed highly similar transcriptional profiles indicative of the activation of multiple inflammatory pathways. This and a related study [62] further showed that in ELANE-SCN, a pro-inflammatory state characterized by elevated IFN responses already exists prior to the acquisition of *CSF3R* and *RUNX1* mutations, which is then stepwise aggravated and involving additional inflammatory pathways (TNF/NFkB, IL-6) upon acquisition of the mutations associated with leukemic progression. These combined observations in mouse and patient models support a link between inflammation to leukemia predisposition and clonal evolution toward MDS/AML in SCN (Fig. 1), similar to what has recently been proposed for MPN and MDS [63,64]. Together, these findings identify inflammation as a common driver for malignant transformation in these conditions, albeit ignited by distinct underlying mechanisms.

Finally, iPSC modeling of specific phases of leukemia progression has identified upregulation of *BAALC* expression as an additional and step in the malignant transformation of SCN toward AML, driven through the phosphorylation of the mitogen-activated protein kinase-activated protein kinase 2a (MK2a) [65^▪^]. Hence, it has been proposed that selective targeting of *BAALC* expression and/or MKa2 phosphorylation may prevent transformation or eliminate AML blasts in SCN/AML [65^▪^].

## CONCLUSION

This review has summarized the recent developments in disease models and gene editing technologies that contributed to the progress made in understanding the biology of SCN and its predisposition to leukemia. Yet, many issues are still unresolved. First, it remains unknown to what extent cell extrinsic (‘niche’) factors contribute to disease development, as has for instance been implicated in SDS, MDS, and MPN [63,66–69]. Second, although excessive oxidative damage, UPR and ER stress responses and ribosomal dysfunction have all been implicated in the development of neutropenia, evidence that these represent targetable vulnerabilities is still modest. With respect to disease progression to MDS/AML, the findings that commonly acquired mutations (*CSF3R*, *RUNX1*, *ASXL1*) and cytogenetic abnormalities (-7, 7q-, trisomy 21) act in concert with elevated inflammation in HSPCs may open avenues for new anti-inflammatory therapies to prevent malignant progression or to eradicate SCN/AML blasts, similar to what has been proposed for MDS and MPN. Likely, the highest expectations for curative therapies should come from the recent developments in gene editing technologies, as for instance currently being clinically explored in sickle cell disease [70].

## Acknowledgements

*I acknowledge members of the Department of Hematology at Erasmus MC and the EuNet-INNOCHRON network for discussions.*

### Financial support and sponsorship

*No financial support was required for this review article.*

### Conflicts of interest

*There are no conflicts of interest.*

## REFERENCES AND RECOMMENDED READING

Papers of particular interest, published within the annual period of review, have been highlighted as:

▪ of special interest
▪▪ of outstanding interest
